# Systematic Review on Fractal Dimension of the Retinal Vasculature in Neurodegeneration and Stroke: Assessment of a Potential Biomarker

**DOI:** 10.3389/fnins.2020.00016

**Published:** 2020-01-28

**Authors:** Sophie Lemmens, Astrid Devulder, Karel Van Keer, Johan Bierkens, Patrick De Boever, Ingeborg Stalmans

**Affiliations:** ^1^Department of Ophthalmology, University Hospitals UZ Leuven, Leuven, Belgium; ^2^Research Group Ophthalmology, Biomedical Science Group, Department of Neurosciences, KU Leuven, Leuven, Belgium; ^3^Health Unit, VITO (Flemish Institute for Technological Research), Mol, Belgium; ^4^Centre of Environmental Sciences, Hasselt University, Diepenbeek, Belgium

**Keywords:** fractal dimension, retina, brain, neurodegeneration, cognitive impairment, Alzheimer's disease, stroke, cerebral small vessel disease

## Abstract

**Introduction:** Ocular manifestations in several neurological pathologies accentuate the strong relationship between the eye and the brain. Retinal alterations in particular can serve as surrogates for cerebral changes. Offering a “window to the brain,” the transparent eye enables non-invasive imaging of these changes in retinal structure and vasculature. Fractal dimension (FD) reflects the overall complexity of the retinal vasculature. Changes in FD could reflect subtle changes in the cerebral vasculature that correspond to preclinical stages of neurodegenerative diseases. In this review, the potential of this retinal vessel metric to serve as a biomarker in neurodegeneration and stroke will be explored.

**Methods:** A literature search was conducted, following the PRISMA Statement 2009 criteria, in four large bibliographic databases (Pubmed, Embase, Web Of Science and Cochrane Library) up to 12 October 2019. Articles have been included based upon their relevance. Wherever possible, level of evidence (LOE) has been assessed by means of the Oxford Centre for Evidence-based Medicine Level of Evidence classification.

**Results:** Twenty-one studies were included for qualitative synthesis. We performed a narrative synthesis and produced summary tables of findings of included papers because methodological heterogeneity precluded a meta-analysis. A significant association was found between decreased FD and neurodegenerative disease, mainly addressing cognitive impairment (CI) and dementia. In acute, subacute as well as chronic settings, decreased FD seems to be associated with stroke. Differences in FD between subtypes of ischemic stroke remain unclear.

**Conclusions:** This review provides a summary of the scientific literature regarding the association between retinal FD and neurodegenerative disease and stroke. Central pathology is associated with a decreased FD, as a measure of microvascular network complexity. As retinal FD reflects the global integrity of the cerebral microvasculature, it is an attractive parameter to explore. Despite obvious concerns, mainly due to a lack of methodological standardization, retinal FD remains a promising non-invasive and low-cost diagnostic biomarker for neurodegenerative and cerebrovascular disease. Before FD can be implemented in clinic as a diagnostic biomarker, the research community should strive for uniformization and standardization.

## Introduction

### Rationale

The retina and optic nerve are outgrowths of the embryonic diencephalon and therefore share anatomic similarities, and functional and immunological characteristics with the brain (London et al., 2013; De Groef and Cordeiro, 2018). Hence, the retina can be approached as an integral part of the central nervous system. The occurrence of ocular manifestations in neurodegenerative and cerebrovascular pathologies, such as Alzheimer's disease (AD), Parkinson's disease (PD) and stroke, accentuates the strong relationship between the eye and the brain (Archibald et al., 2009; Armstrong, 2015; Lim et al., 2016; Cheung et al., 2017; Mahajan and Votruba, 2017). Retinal changes in particular can present a surrogate for cerebral changes in these disorders. Offering a “window to the brain,” the transparent eye enables non-invasive imaging of these changes in retinal structure and vasculature.

Microvascular changes are an important component of neurodegenerative and cerebrovascular diseases (Iadecola, 2010; Brown and Thore, 2011; Wardlaw et al., 2013; Shi and Wardlaw, 2016). The presence of a retinal counterpart of these vascular changes has been reported repeatedly (Patton et al., 2007; Frost et al., 2013; Cheung et al., 2019). Fundus photography is a routine analysis to visualize retinopathy signs (retinal hemorrhage, microaneurysms) and retinal vascular caliber changes, which have been shown to be associated with these disorders of the central nervous system. Central retinal arteriolar equivalent (CRAE) and central retinal venular equivalent (CRVE) quantify generalized retinal vessel narrowing or widening, matching subtle dysfunction of the retinal microvasculature. Decreased CRVE and CRAE have been associated with AD (Frost et al., 2013; Cheung et al., 2014b). But the retinal vascular tree holds more information than these focal measurements, and advances in retinal imaging and (semi-)automated image processing and analysis rose scientific interest in the association between retinal vessel metrics such as the fractal dimension (FD), tortuosity and branching of the retinal vascular network and neurodegenerative and cerebrovascular disorders. These retinal vascular network parameters quantify global vessel network characteristics, reflecting the integrity of the cerebral microcirculation (Cheung et al., 2015).

The FD of the retinal vascular network is a relatively novel parameter, introduced by Mandelbrot and Wheeler (1983) that has already been described in many subfields of medicine (Mandelbrot and Wheeler, 1983; Goldberger and West, 1987; Stanley et al., 2005). In 1990, Mainster was the first to describe the retinal microvascular network as a fractal, implying great potential to gain new insights in the complex arborization pattern of the retinal vascular network (Mainster, 1990). During the last three decades, this upcoming non-invasive parameter has proven its merits in various research papers often covering multidisciplinary research combining ophthalmology with cardiology, endocrinology, or neurology. Significant correlations with refractive error (Li et al., 2010; Al-Sheikh et al., 2017), glaucoma (Kolár and Jan, 2008; Wu et al., 2013), age-related macular degeneration (Al-Sheikh et al., 2018), cardiovascular health (Liew et al., 2008; Cheung et al., 2011) and diabetes mellitus have been reported (Avakian et al., 2002; Cheung et al., 2009). In 2019 alone, more than 20 research papers addressing the retinal FD, have been registered in MEDLINE. The FD of the retinal vascular network is a measure of its complexity and vessel density (Mainster, 1990; Misson et al., 1992; Masters, 2004; Kwa and Lopez, 2010; Ab Hamid et al., 2016). The retinal vasculature is a transport network delivering oxygen and nutrients to the retinal tissue and removing waste products. This network strives for minimal energy consumption, reflected by its accordance to Murray's Law of Minimal Work, which describes an optimal relationship between the radii of mother and daughter branches in a network. This law leads to the typical vessel arborization pattern of the retina (Rossitti, 1995). Many parameters of this vascular tree are being studied, and FD is one expressing the density and overall complexity of this spatial pattern in one number. It literally means “broken dimension.” The retinal microvasculature can be considered to be more dimensional than a line (1-dimensional), but less than a square (2-dimensional), hence a dimension between 1 and 2 can be attributed to this pattern with a higher number reflecting a more complex branching pattern. The retinal FD is associated with age (Azemin et al., 2012; Che Azemin et al., 2013; Wei et al., 2017), refractive error (Li et al., 2010, 2017; Azemin et al., 2014; Yang et al., 2016; Al-Sheikh et al., 2017; Tai et al., 2017) and comorbidities (Avakian et al., 2002; Liew et al., 2008; Cheung et al., 2009, 2011).

In our review we collect and summarize the current literature on FD measurements from studies addressing possible associations between global retinal parameters and cerebral disease, with a particular focus on dementia and stroke.

### Objective

This systematic review aims to provide an overview of the current scientific evidence of changes in the FD of the retinal vasculature in subjects affected by central neurodegenerative disease or stroke.

### Research Question

Is the retinal FD significantly altered in patients with central neurodegenerative disease and/or stroke and to what extent can retinal FD serve as a non-invasive biomarker for these diseases?

What could be the potential future applications of retinal FD and which hurdles are still to be overcome?

## Methods

We adopted the Preferred Items for Systematic Reviews and Meta-Analysis (PRISMA) guidelines (Moher et al., 2009).

### Search Strategy

We searched: MEDLINE (PubMed), EMBASE (Ovid), Web Of Science (Clarivate Analytics), and Cochrane Library (Cochrane). Search strategies were constructed using database specific subject headings and keywords. The search strategies are provided as (Supplementary Data 1). These searches were extended by hand searching the bibliographies of all included studies.

Gray literature was not considered. Accepted language of publication were: English, German, French and Dutch. Articles published until October 12, 2019 were included.

### Study Design

Case-control studies, cohort studies, and case series were included. Case reports, case series with < 10 patients, reviews and articles without original results were excluded.

### Participants, Interventions, Comparators

We included studies on patients affected by stroke or central neurodegenerative disease.

Severity of stroke, etiology of stroke, stages of cognitive impairment (CI), or etiology of central neurodegeneration were not exclusion criteria.

We included studies using the following retinal imaging techniques:

- Conventional digital fundus photography
- Scanning laser ophthalmoscopy (SLO)
- Optical coherence tomography angiography (OCT-A).

### Data Sources, Studies Sections, and Data Extraction

According to the PRISMA flow diagram, screening of titles and abstracts was carried out. Non-pertinent articles were rejected. Duplicates were removed using Mendeley Reference Manager (by Mendeley, London, UK). After this initial selection, full texts were independently judged for eligibility by three independent reviewers (SL, AD, JB) and inconsistencies were solved by consensus. We used the Oxford Centre for Evidence-based Medicine classification to determine the LOE (Phillips, 2014) of the papers included in this qualitative synthesis (Table 1).

**Table 1:** Demographic and clinical characteristics.

**Author, year**	**Pathology**	**N patients**		**Age (years) Mean** **±** **SD**		**Sex M/F (%)**		**Study design (LOE)**	**Clinical characteristics**
		**CA**	**CO**	**CA**	**CO**	**CA**	**CO**		
Aliahmad et al. (2014)	STROKE	46	39	67.6 ± 5.8	67.9 ± 5.6	54/46	54/46	Case-control (3b)	Stroke cases and controls from the BMES cohort. Diagnosis based on World Health Organization Monitoring Trends and Determinants in Cardiovascular Disease (WHO-MONICA) + CT or MRI
Cabrera DeBuc et al. (2018)	CI	20	19	81 ± 6	80 ± 7	20/80	16/84	Case-control (3b)	CI cases and controls recruited in a non-systematic fashion as they appeared in the clinic or identified from a population attending adult care centers and community clinics. Diagnosis of CI according to MoCA. No CI staging (including AD cases)
Cavallari et al. (2011)	CADASIL (STROKE)	10	10	43.8	43.5	40/60	40/60	Case series (4)	CADASIL cases from 5 Italian families. Diagnosis of CADASIL by molecular diagnosis
Cheung et al. (2010)	STROKE	392 (45 LI)		67.2 ± 14.1		57/43		Cohort (2b)	Acute lacunar stroke patients from 2 stroke centers in Australia. Diagnosis of lacunar infarction (LI) based on CT and/or MRI
Cheung et al. (2013)	STROKE	2644		57.4 ± 10.7		48/52		Cohort (2b)	Data derived from the Singapore Malay Eye study (SiMES). During follow-up, 51 participants had an incident stroke event. Confirmation by record linkage with the stroke cases registered by National Registry of Diseases Office, Singapore (electronically captured and compulsory by law)
Cheung et al. (2014a)	CI	1202		70.6 ± 5.4	67.9 ± 5.2	21/79	59/41	Cohort (2b)	Data derived from the SiMES. Diagnosis of CI based on AMT score. Of the 1202 participants, 262 (21.8%) had cognitive dysfunction as defined by the AMT with education-adjusted cutoffs. No CI staging
Cheung et al. (2014b)	AD	136	290	74.8 ± 5.7	73.9 ± 4.6	47/53	53/47	Case-control (3b)	AD cases from three tertiary hospitals in Singapore. Controls from population-based studies under the Singapore Epidemiology of Eye Disease (SEED) program, which includes the Singapore Chinese Eye Study (SCES), the Singapore Indian Eye Study (SINDI), and SiMES. Diagnosis of dementia syndrome according to DSM-IV, diagnosis of AD according to NINCDS-ADRDA, inclusion of controls based on AMT score
Csincsik et al. (2018)	AD	56 baseline/9 follow-up	48 baseline/14 follow-up	79.2 ± 8.4	70.7 ± 10.4	NS	NS	Case-control (3b)	AD cases and controls from the West London Cognitive Disor-ders Treatment and Research Unit (WLCDTRU). Controls from carers of index patients. Diagnosis of AD according to NINCDS-ADRDA criteria. Follow-up after 2 years
Doubal et al. (2010)	STROKE	86	80	65 ± 11	69 ± 11	63/37	70/30	Case-control (3b)	Cases (acute lacunar stroke) and controls (mild cortical stroke) from UK hospital stroke service, Mild Stroke Study (MSS). Assessment of stroke severity using the National Institutes of Health Stroke Scale and classification of the stroke clinical syndrome (lacunar or cortical) according to the Oxfordshire Community Stroke Project classification as well as using radiologic criteria (MRI)
Frost et al. (2013)	AD	25	(1) 123 (2) 30 Ab–, 15 Ab+	72.4 ± 7.5	(1) 71.6 ± 5.6 (2) 70.4 ± 5.3, 73.7 ± 6.3	48/52	(1) 45/55 (2) 50/50, 60/40	Case-control (3b)	AD cases and controls from the Australian Imaging, Biomarkers and Lifestyle (AIBL) study of ageing. Diagnosis of probable AD according to NINCDS-ADRDA criteria. Two study components: (1) ‘clinical status study': retinal vascular parameters (RVP) differences between 25 AD and 123 CO participants, and (2) “neuroimaging study”: RVP with respect to neocortical plaque burden in CO participants with AIBL neuroimaging data available (*n =* 45)
Hilal et al. (2014)	CSVD in CI	261 (36 LI−29 WML - 83 CMB)		70.0 ± 0.4		47/53		Cohort (2b)	Chinese participants from Epidemiology of Dementia in Singapore (EDIS) study aged ≥60 years were screened using AMT and self-report of progressive forgetfulness. Diagnosis of cerebral small vessel disease (CSVD) based on MRI. No CI staging
Jiang et al. (2018)	MCI + AD	12 AD19 MCI	21	73.3 ± 9.6 69.6 ± 9.8	67.6+/8.3	58/42 37/63	33/67	Case-control (3b)	MCI + AD cases from the McKnight Brain Registry and referred from the Division of Cognitive Disorders at the University of Miami to the neuroophthalmology clinic at the Bascom Palmer Eye Institute. Recruitment of controls: NS. Diagnosis of AD and MCI based on National Institute on Aging–Alzheimer's Association (NIA-AA) criteria
Jung et al. (2019)	ADCI + SVCI	29 Ab+ ADCI28 Ab– SVCI	34	73.8 ± 8.0 75.4 ± 8.0	69.8 ± 6.1	45/55 29/71	21/79	Case-control (3b)	CI cases and controls from Samsung Medical Center, Republic of Korea. 29 Alzheimer's disease CI (ADCI): 6 amnestic MCI (aMCI) and 23 probable AD dementia. 28 subcortical vascular CI (SVCI): 17 subcortical vascular MCI (svMCI) and 11 subcortical vascular dementia (SVaD). Probable AD dementia according to NINCDS-ADRDA). SVaD according to DSM-IV) and imaging criteria for SVaD proposed by Erkinjuntti et al. aMCI and svMCI patients met Petersen's criteria for MCI with modifications. All svMCI and SVaD patients had severe WMH on MRI scans
Kawasaki et al. (2011)	STROKE	101	184	73.8 ± 8.2 (age-matched)		42/58 (gender-matched)		Case-control (3b)	Stroke cases and controls from the BMES cohort. Diagnosis based on WHO-MONICA + CT or MRI
McGrory et al. (2019)	CSVD/ STROKE	603		72.5 ± 0.7		50/50		2 cohorts (2b)	Lower burden of CSVD. Participants from second wave of testing in Lotharian Birth Cohort 1936 (LBC1936) study. 84 with any history of stroke: 22 self-reported, 62 imaging evidence.
		155		66.9 ± 11.4		68/32			Higher burden of CSVD. Participants from MSS: prospective study of patients with recent (within 3 months) clinical lacunar or mild cortical ischemic stroke. Participants from UK hospital stroke service. Assessment of stroke severity using the National Institutes of Health Stroke Scale and classification of the stroke clinical syndrome (lacunar or cortical) according to the Oxfordshire Community Stroke Project classification as well as using radiologic criteria (MRI)
Naidu et al. (2016)	CIND in T2DM	69	68	Range: 18–75	Range: 18–75	45/55	65/35	Case-control (3b)	Cases and controls from the South London Diabetes Study (SOUL-D), an ongoing prospective study of people with newly diagnosed type 2 diabetes (T2DM). Diagnosis of CI based on modified Telephone Interview for Cognitive Status (TICSM). Cases: TICSM scores in the lowest 10% of the sample distribution (score 17 or below), controls: randomly selected sample of remaining participants. Dementia cases excluded.
Ong et al. (2013)	STROKE	557	557	61.9 ± 9.4	61.9 ± 9.1	64/36	64/36	Case-control (3b)	Cases from one study site of the Multi-Centre Retinal Stroke (MCRS) study (Singapore General Hospital, Singapore), with first-ever or recurrent ischemic stroke (261 lacunar, 185 large artery, 54 cardioembolic), within 7 days of onset. Controls from participants of the Singapore Epidemiology of Eye Diseases (SEED) study. Diagnosis of stroke based on clinical neurological assessment + CT or MRI. Stroke classification based on modified version of the Trial of Org 10172 in Acute Stroke Treatment (TOAST) classification.
Ong et al. (2014)	CIND	78 CIND-mild 69 CIND-mod	121 NCI	71.1 ± 6.3 74.1 ± 5.4	67.3+/4.8	46/54 32/68	44/56	Cohort (2b)	Chinese participants from EDIS study aged ≥60 years were screened for CI using AMT and self-report of progressive forgetfulness. All 268 subjects included were screening-positives. Definite classification was based on detailed neuropsychological testing and MRI. Non-cognitive impairment (NCI) was diagnosed if participants were not impaired in any of the domains tested. CIND was defined as impairment in 1 or more domains in the neuropsychological test battery. CIND-mild was diagnosed if 1 or 2 domains were impaired, and CIND-moderate if more than 2 domains were impaired
Shi et al. (2019)	PD	25	25	61.9 ± 7.6	59.0 ± 5.8	52/48	52/48	Case-control (3b)	PD cases from Neurology Department of Wenzhou People's Hospital, China. Controls from working staff at the Eye Hospital of Wenzhou Medical University, China. Diagnosis of PD based on United Kingdom Brain Bank Criteria, recording of disease severity (Hoehn and Yahr scale), disease duration, and treatment.
Taylor et al. (2015)	Cognitive ability in physiological aging	648		72.4 ± 0.71		50/50		Cohort (2b)	Participants from LBC1936 study. Individuals with a MMSE score <24 were excluded (cut-off to exclude individuals with possible dementia)
Williams et al. (2015)	AD	213	294	79.6 ± 7.8	76.3 ± 6.6	36/64	40/60	Case-control (3b)	Cases from opportunistic screening in hospital memory clinic, United Kingdom. Controls from caretakers of patients attending any out-patient clinic in the study hospital, university press release, controls asked friends to participate, and a series of talks given to AD patient support groups in the region led to volunteers. Diagnosis of AD according to NINCDS-ADRDA criteria

*Ab+, beta-amyloid positive; Ab-, beta-amyloid negative; AD, Alzheimer's disease; ADCI, Alzheimer's disease cognitive impairment; AIBL, Australian Imaging; Biomarkers and Lifestyle; aMCI, amnestic mild cognitive impairment; AMT, Abbreviated Mental Test; BMES, Blue Mountains Eye Study; CA, case; CADASIL, cerebral autosomal dominant arteriopathy with subcortical infarcts and leukoencephalopathy; CI, cognitive impairment; CIND, cognitive impairment no dementia; CMB, cerebral microbleeds; CO, control; CSVD, cerebral small vessel disease; CT, computed tomography; DSM-IV, Diagnostic and Statistical Manual of Mental Disorders IV; EDIS, Epidemiology of Dementia in Singapore; LBC1936, Lotharian Birth Cohort 1936; LI, lacunar infarction; LOE, level of evidence; M/F, male-female ratio; MCI, mild cognitive impairment; MCRS, Multi-Centre Retinal Stroke; MMSE, Mini-Mental State Examination; MoCA, Montreal Cognitive Assessment; MRI, magnetic resonance imaging; MSS, Mild Stroke Study; NCI, noncognitive impairment; NIA-AA, National Institute on Aging–Alzheimer's Association; NINCDS-ARDRA, National Institute of Neurological and Communicative Disorders and Stroke and the Alzheimer's Disease and Related Disorders Association; PD, Parkinson's disease; RVP, retinal vascular parameters; SCES, Singapore Chinese Eye Study; SD, standard deviation; SEED, Singapore Epidemiology of Eye Disease; SiMES, Singapore Malay Eye Study; SINDI, Singapore Indian Eye Study; SVaD, subcortical vascular dementia; SVCI, subcortical vascular cognitive impairment; svMCI, subcortical vascular mild cognitive impairment; T2DM, type 2 diabetes mellitus; TICSM, Telephone Interview for Cognitive Status; TOAST, Trial of Org 10172 in Acute Stroke Treatment; WHO-MONICA, World Health Organization Monitoring Trends and Determinants in Cardiovascular Disease; WLCDTRU, West London Cognitive Disor-ders Treatment and Research Unit*.

### Data Analysis

The main outcome of this systematic review was the current understanding of changes in the FD of the retinal vasculature in central neurodegenerative disease and stroke patients. Results were separately analyzed and presented for studies addressing stroke and neurodegenerative pathology. In the subset of central neurodegeneration studies, differences between various types and stages of CI were analyzed wherever possible.

## Results

### Study Selection

**Figure 1:**
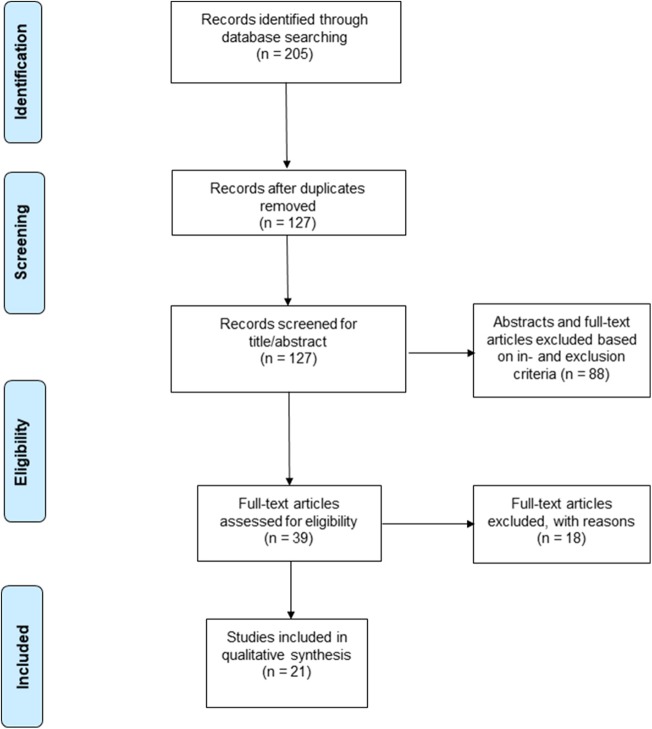
PRISMA 2009 flow diagram (Moher et al., 2009).

A total of 205 studies were screened using the described search strategy. At the end of the selection process, 13 case-control studies, seven cohort studies and one case series were included in the systematic review. Prisma flow diagram (Figure 1) gives details on screening process. Six out of 21 (29%) studies examined retinal vascular changes in terms of FD alone, whereas the 15 (71%) remaining studies examined at least one other vascular metric.

### Study Characteristics

A total of 9,397 subjects were considered in this systematic review. Nine studies included stroke patients, 12 included neurodegeneration patients, with one of these including patients with disease overlap between stroke and neurodegeneration. Because of the clinical characteristics of the study population (CI subjects, based on Abbreviated Mental Test (AMT) score and self-report of progressive forgetfulness), this study by Hilal et al. has been included in the neurodegenerative section of this review (Hilal et al., 2014).

Regarding studies focusing on neurodegenerative disease, four included only patients with cognitive impairment no dementia (CIND), four had only patients with AD, two combined CIND as well as dementia patients, one included only patients with PD, and finally one study reported on the parameters of the retinal microvascular network and cognitive ability in physiological aging in a cohort of older adults without CI. Severity of CI with or without dementia varies from mild to severe and some authors included different stages in the same study. However, the method of severity classification was not consistent throughout all studies.

Stroke subtype was not specified in three out of eight stroke studies and in a subset of 84 out of 231 stroke patients belonging to a fourth study (the latter corresponding with 15% of the total number of stroke patients). Three studies included different types of ischemic stroke whereas two studies included lacunar stroke patients only.

Patient characteristics of included studies are reported in Table 1. Subjects included in the central neurodegeneration section of this review show heterogeneity in mean age ranging from 59 (Shi et al., 2019) up to 81 (Cabrera DeBuc et al., 2018) years. In the section dedicated to studies focusing on stroke patients, the heterogeneity in mean age is even higher, from 44 (Cavallari et al., 2011) up to 74 (Kawasaki et al., 2011). Most studies accounted for potential confounding due to age, gender and cardiovascular risk factors by multivariate regression or exclusion. The effect of refractive error and ocular comorbidities however, was rarely described, let alone taken into account in statistical analysis.

### Outcomes of Fractal Analysis in Studies on Neurodegenerative Disease

Changes in the FD of the retinal vasculature in neurodegenerative disease patients are reported in Table 2. One particular methodological set-up was the strategy of choice in eight out of 13 neurodegeneration studies. These studies combined their focus on CI and/or dementia with a region of interest (ROI) of 0.5–2.0 disc diameters from the disc margin on a disc-centered fundus image, taken with a conventional 45° field-of-view (FOV) digital retinal camera and processed and analyzed using the commercially available Singapore I Vessel Assessment (SIVA, National University of Singapore, Singapore) software, offering curvature-based vessel segmentation and calculation of the FD as a monofractal using the box counting method. Seven of these papers reported a decreased FD in AD (Frost et al., 2013; Cheung et al., 2014b; Williams et al., 2015), CI (including dementia) (Cheung et al., 2014a), subcortical vascular CI (including vascular dementia) but not in CI related to AD (Jung et al., 2019), or CIND (Ong et al., 2014), as opposed to cognitively normal controls. Lower FD was also reported in cognitively impaired elderly with multiple cerebral microbleeds, as opposed to cognitively impaired elderly without multiple cerebral microbleeds (Hilal et al., 2014). Naidu et al. applied the same strategy in a population of recently diagnosed type 2 diabetics with and without CIND, but found no association between FD and cognitive status (Naidu et al., 2016). Some of the studies using this method showed an association between FD and AMT score (Cheung et al., 2014a) and cognitive performance (Ong et al., 2014), but a significant relationship between FD and Mini-Mental State Examination (MMSE) (Jung et al., 2019) or plaque burden in cognitively normal controls (Frost et al., 2013) could not be confirmed.

**Table 2:** Outcomes of studies focusing on neurodegenerative pathology and cognition.

**Author, year**	**RVP(s) analyzed**	**Imaging device**	**FD ROI(s)**	**FD calculation**	**Results FD**
Cabrera DeBuc et al. (2018)	**FD0, FD1, FD2 (multifractal)** CRAE, CRVE cTORTa, cTORTv BCa, BCv AFa, AFv LDRa, LDRv AVR [Also: electroretinography, color vision quantification, visual performance test]	45° digital SLO camera (EasyScan, iOptics, Netherlands)	Whole area of visible retina permitted by each image (from the OD boundary out toward the periphery)	SIVAImageJ (http://rsb.info.nih.gov/ij) together with the FracLac plug-in was used to calculate the multifractal properties of the retinal vasculature network	The vascular FD, assessed with a multifractal approach, was lower in individuals with CI (capacity, information and correlation dimensions: FD0, FD1, and FD2) vs. controls
Cheung et al. (2014a)	**FDt (monofractal)** CRAE, CRVE cTORTa, cTORTv BAa, BAv	45° digital retinal camera (Canon CR-DGi with a 10D SLR digital camera back; Canon, Japan)	0.5–2.0 DD from disc margin	SIVA	Participants with lower retinal vascular FD values were more likely to have cognitive dysfunction
Cheung et al. (2014b)	**FDt, FDa, FDv (monofractal)** CRAE, CRVE cTORTa, cTORTv BAa, BAv	45° digital retinal camera (Canon CR-DGi 10D or Canon CR-1 40D; Canon, Japan)	0.5–2.0 DD from disc margin	SIVA	Compared with the normal controls, the AD patients had smaller total and FDa. Persons with smaller FDt, FDa and FDv were more likely to have AD, controlling for potential confounders. These associations were still persistent after only AD cases without history of cerebrovascular disease were included
Csincsik et al. (2018)	**FDa, FDv (monofractal)** WGa, WGv TORTa, TORTv (algorithm NS) … (not explicitly stated) [Also: peripheral drusen]	Optomap P200C UWF-SLO (Optos Plc, Dunfermline, UK)	0.5–1.0 DD from the disk margin 0.5–2.0 DD from the disk margin Whole area of visible retina permitted by each image (from the OD boundary out toward the periphery)	Automated segmentation of the vasculature by algorithm developed by Pellegrini et al. (2014). Manual refinement removed artefacts (i.e., spurious vessel detections) and separated out the arteriolar and venular components of the vascular tree by labeling vessels and marking crossing points by hand. RVPs were measured using software specially designed to handle UWF imaging, VAMPIRE for UWF-SLO (v1, Universities of Edinburgh and Dundee, United Kingdom)	There was a significant decrease in FDa in AD at baseline with a trend at FU. The most consistent differences between AD patients and controls were observed when the entire image was considered
Frost et al. (2013)	**FDa, FDv (monofractal)** CRAE, CRVE AVR BSTDa, BSTDv cTORTa, cTORTv Num1stBa, Num1stBv BCa, BCv AFa, AFv JEa, JEv LDRa, LDRv	45° digital retinal camera (Canon CR-1 non-mydriatic camera, Canon USA, Lake Success, NY, USA)	0.5–2.0 DD from the disk margin	SIVA	Reduced complexity of the branching pattern in AD (FD, Num1stB). No association between FD and (high) plaque burden in healthy individuals
Hilal et al. (2014)	**FDa, FDv (monofractal)** CRAE, CRVE cTORTa, cTORTv	45° digital retinal camera (Canon CR-DGi 10D or Canon CR-1 40D; Canon, Japan)	NS (most likely 0.5–2.0 DD from disc margin)	SIVA	Smaller FDa was associated with presence of multiple CMB. No association was found with lacunar infarcts and WML volume. After multivariate adjustments, association remained statistically significant
Jiang et al. (2018)	**FDrvn**, **FDsvp**, **FDdvp (monofractal)** Representing microvascular density [Also: GC-IPL thickness]	Zeiss Angioplex OCTA (Carl Zeiss Meditec, Dublin, CA), covering a retinal area of 3 x 3 mm^2^ centered on the fovea	The area between circles centered on the fovea and diameters of 0.6–2.5 mm was defined as the annular zone. The annular zone was then divided into 4 quadrantal sectors, named the superior temporal, inferior temporal, superior nasal, and inferior nasal. The annular zone was also divided into 6 thin annuli with a width of ~0.16 mm (C1-C6). Fractal analysis was performed in each sector or annular zone.	Automated segmentation of the vasculature by algorithm developed by Jiang et al. (2013). Fractal analysis was performed in each sector or annular zone using the box counting method with the fractal analysis toolbox (Benoit, Trusoft Benoit Fractal Analysis Toolbox; Trusoft International, Inc, St. Petersburg, USA).	Patients with AD had lower densities of RVN, SVP, and DVP in the annular zone, in comparison with controls. Patients with MCI had lower density of DVP in the superior nasal quadrant than that of the controls. There was a trend of vascular density loss from control to MCI then AD
Jung et al. (2019)	**FDt, FDa, FDv (monofractal)** CRAE, CRVE BAa, BAv	45° digital retinal camera (TRC-50DX; Topcon Medical Systems, Inc., USA)	0.5–2.0 DD from disc margin	SIVA	Compared to NCI individuals, the SVCI patients had smaller FDt and FDa, whereas there was no significant difference of FD between ADCI and NCI
Naidu et al. (2016)	**FDt, FDa, FDv (monofractal)** CRAE, CRVE AVR sTORTt, sTORTa, sTORTv, cTORTt, cTORTa, cTORTv	45° digital retinal camera Topcon Fundus Camera (TRC 50-VT; Tokyo Optical, Tokyo, Japan)	0.5–2.0 DD from disc margin	SIVA	No significant differences between cases and controls in FD
Ong et al. (2014)	**FDa, FDv (monofractal)** CRAE, CRVE cTORTa, cTORTv	Non-mydriatic digital camera (NS)	0.5–2.0 DD from disc margin	SIVA	Reduced retinal FDa and FDv were associated with an increased risk of CIND-mild and CIND-moderate. Reduced FD was associated with poorer cognitive performance globally and in the specific domains of verbal memory, visuoconstruction and visuomotor speed
Shi et al. (2019)	In annular zone + per quadrant: **FDsvp**, **FDdvp (monofractal)** retinal capillary perfusion density Retinal capillary skeleton density	CommercialSD-OCT system (Optovue RTVue XR Avanti; Optovue, Inc, Fremont, CA, OCT-A images derived from the built-in software [Angiovue, Version 2015.1.90)], covering a retinal area of 3 x 3 mm^2^ centered on the fovea	The area between circles centered on the fovea and diameters of 0.6–2.5 mm was defined as the annular zone. The annular zone was then divided into 4 quadrantal sectors, named the superior, inferior, nasal, and temporal.	FD was calculated based on skeletonized images of the retinal capillary network in the OCT-A images, using the following series of image processing procedures to create binary images of the vessels: bicubic interpolation, segmentation, detection of the FAZ boundary, and determination of the background signal-to-noise ratio. Fractal analysis software (Benoit, Trusoft Benoit Fractal Analysis Toolbox; Trusoft International, Inc, St. Petersburg, USA) was applied to the image analysis	The superficial retinal capillary plexus in PD patients had lower capillary complexity in the total annular zone and all quadrant sectors compared with healthy control subjects. The deep retinal capillary plexus complexity was decreased in the total annular zone and the superior and inferior quadrants. The retinal capillary complexity in the inferior quadrant was negatively correlated with the best-corrected visual acuity and disease duration
Taylor et al. (2015)	**FDt (monofractal)** **FD0, FD1, FD2 (multifractal)**	45° digital retinal camera (CRDGi; Canon USA Inc., Lake Success, NY)	Whole area of visible retina permitted by each image (from the OD boundary out toward the periphery)	Automated segmentation of the retinal microvascular network was performed using an algorithm described previously by Soares et al. (2006). Images were analyzed by an expert retinal image analyst using VAMPIRE custom software (School of Computing, University of Dundee, United Kingdom) built in Matlab (The MathWorks, Natwick, MA)	Only three out of 24 comparisons were found to be significant. No association survived Bonferroni correction for multiple statistical testing. Significant unadjusted associations were weakened and lost significance after covarying for IQ at age 11 and cardiovascular risk factors, and not one association was verified by an equivalent finding using measurements from the contralateral eye
Williams et al. (2015)	**FDt, FDa, FDv (monofractal)** CRAE, CRVE cTORTa, cTORTv BAa, BAv	45° digital retinal camera (500 Canon CR-DGi; Canon, Japan)	0.5–2.0 DD from disc margin	SIVA	AD patients have a sparser retinal microvascular network with significantly lower FDt, FDa and FDv. Subjects with lower FDv were more likely to have AD. A secondary analysis failed to detect any significant associations between retinal microvascular parameters and MMSE score

*AD, Alzheimer's disease; ADCI, Alzheimer's disease cognitive impairment; AFa/v, arteriolar/venular asymmetry factor; AVR, artery-vein ratio; BAa/v, arteriolar/venular branching angle; BCa/v, arteriolar/venular branching coefficient; BSTDa/v, zone B standard deviation of arteriolar/venular width; CRAE, central retinal arteriolar equivalent; CRVE, central retinal venular equivalent; CI, cognitive impairment; CIND, cognitive impairment no dementia; CMB, cerebral microbleeds; cTORTa/v, curvature arteriolar/venular tortuosity; DD, disc diameter; DVP, deep vascular plexus; FAZ, foveal avascular zone; FD, fractal dimension; FDt/a/v, total/arteriolar/venular fractal dimension; FD0, capacity dimension; FD1, information dimension; FD2, correlation dimension; IQ, intelligence quotient; JEa/v, junctional exponent deviation for arterioles/venules; LDRa/v, arteriolar/venular length-diameter ratio; MCI, mild cognitive impairment; MMSE, Mini-Mental State Examination; NCI, noncognitive impairment; NS, not specified; Num1stBa/v, number of first branching arterioles/venules; OCT-A, optical coherence tomography angiography; OD, optic disc; PD, Parkinson's disease; RVN, retinal vascular network; ROI, region of interest; SD-OCT, spectral domain optical coherence tomography; SIVA, Singapore I Vessel Assessment; SLO, scanning laser ophthalmoscopy; sTORTa/v, simple arteriolar/venular tortuosity; SVCI, subcortical vascular cognitive impairment; SVP, superficial vascular plexus; UWF, ultra-widefield; VAMPIRE, Vessel Assessment and Measurement Platform for Images of the REtina; WGa/v, arteriolar/venular width gradient; WML, white matter lesions*.

The five remaining studies used a different methodology. Three of these focused on CI, and even with differences in ROI, imaging device, vessel segmentation software, FD calculation method and software, all three confirmed a significant decrease of FD measures in CI (including dementia) (Cabrera DeBuc et al., 2018), mild CI (Jiang et al., 2018) and AD (Csincsik et al., 2018). After a follow-up period of 2 years, Csincsik et al. reported that the significant difference in FD between AD and controls at baseline had become a trend (Csincsik et al., 2018), whereas Jiang et al. described a trend of vascular density loss from controls over MCI to AD cases (Jiang et al., 2018). A significant association between fractal dimension and Montreal Cognitive Assessment (MoCA) could not be confirmed (Cabrera DeBuc et al., 2018).

The only study including only healthy elderly could not establish an association between FD and cognitive ability, both measured in later life. Differences in childhood cognitive ability appeared to account for much of the variance in cognitive ability in older age (Taylor et al., 2015).

Research by Shi et al., unique in its position addressing retinal microvascular changes in PD and one of two studies using OCT-A, showed that FD of the superficial vascular plexus was decreased in all analyzed regions and that FD of the deep vascular plexus was decreased in all but two ROIs. Of note, this was the only study able to confirm a significant correlation with disease duration (Shi et al., 2019).

### Outcomes of Fractal Analysis in Stroke Studies

Table 3 summarizes the findings on fractal dimension analysis of the retinal vasculature in stroke patients. The eight studies that were kept for our review are heterogeneous, both in methods and in outcome. Cheung et al. and Ong et al. shared the same methodological set-up and analyzed monofractal FD in ischemic stroke patients using SIVA software. Ong et al. reported a significant association between decreased FD and acute ischemic stroke (Ong et al., 2013). Cheung et al. concluded that retinal imaging may help in the discrimination between subjects with a history with and without stroke, as well as stratification of stroke risk (Cheung et al., 2013). Two other studies confirmed a decrease in FD is associated with higher risk of stroke (Kawasaki et al., 2011; Aliahmad et al., 2014). It has to be noted that both studies analyzed data from subsets of the same Blue Mountains Eye Study (BMES) cohort, with the stroke cases included by Aliahmad et al. all being part of those included by Kawasaki et al.

**Table 3:** Outcomes of studies focusing on stroke.

**Author, year**	**RVP(s) analyzed**	**Imaging device**	**FD ROI(s)**	**FD calculation**	**Results FD**
Aliahmad et al. (2014)	**FDCt (monofractal) SFDt (monofractal)** **BCFDt (monofractal)**	30° digital retinal camera (Zeiss FF3 fundus camera)	Three concentric zones: zone A: 0.0–0.5 DD from disc margin zone B: 0.5–1.0 DD from disc margin zone C: 1.0–1.5 DD from disc margin FDC was calculated for all seven possible combinations of the zones: A, B, C, AB, BC, AC, and ABC. SFD and BCFD were calculated for the entire ROI, being zone ABC.	Automated segmentation of the retinal microvascular network was performed using an algorithm described previously by Soares et al. (2006). Software for FD calculation is not specified; information regarding the mathematical approach for the different types of FD calculation is available.	Cases and controls do not differ in a statistically significant way based upon the FDCtA, FDCtC or FDCtAC alone. However, FDCtB, FDCtAB, FDCtBC, and FDCtABC are found to give rise to statistically significant differences. FDCt(ABC) was revealed as a better predictor of stroke compared with SFDt(ABC) and BCFDt(ABC), with overall lower median value for cases compared to controls
Cavallari et al. (2011)	**FDt (monofractal)**	NS	0.0–1.75 DD from disc center	ImageJ (http://rsb.info.nih.gov/ij) together with the FracLac plug-in was used to calculate the fractal properties of the retinal vasculature network.	The results showed that mean-FDt value of cases was lower than in controls. Mean-FDt did not correlate with disease duration nor with MRI lesion volumes of the subjects with CADASIL
Cheung et al. (2010)	**FDt (monofractal)**	45° digital retinal camera (Canon D60, Canon, Tokyo, Japan)	0.0-1.75 DD from disc center	IRIS–Fractal (non-linear orthogonal projection segmentation; Zhang et al., 2009)After the program automatically traced all retinal vessels within this region, the grader checked the tracing with the original photograph and removed occasional artifacts misidentified as vessels (peripapillary atrophy, choroidal vessels, pigment abnormalities, and nerve fiber reflection). The program then performed fractal analysis and calculated retinal FD using the box-counting approach	Higher retinal FDt was independently and positively associated with lacunar stroke
Cheung et al. (2013)	**FDt (monofractal)** CRAE, CRVE cTORTa, cTORTv BAa, BAv [Also: retinopathy signs on fundus photographs]	45° digital retinal camera (Canon CR-DGi with a 10D SLR digital camera back, Canon, Japan)	0.5–2.0 DD from disc margin	SIVA	Retinal imaging improves the discrimination and stratification of stroke risk beyond that of established risk factors by a significant but small margin: compared with the model with only established risk factors, the addition of retinal measures improved the prediction of stroke and correctly reclassified 5.9% of participants with incident stroke and 3.4% of participants with no incident stroke. Whereas, retinopathy signs and larger CRVE were associated with an increased risk of stroke, FDt alone was not significantly associated with a higher risk of stroke
Doubal et al. (2010)	**FDt (monofractal)** **FD0 (multifractal)**	45° digital retinal camera (Canon CR-DGi, Canon USA Inc.)	45° FOV area	Retinal images were analyzed in Matlab (The MathWorks, Natwick, MA), combining fractal analysis with an automatic vessel segmentation procedure	Decreased FDt and FD0 (both representing decreased branching complexity) were associated with increasing age and lacunar stroke subtype
Kawasaki et al. (2011)	**SFDt (monofractal)**	30° digital retinal camera (Zeiss FF3 fundus camera)	0.0–1.25 DD from disc center	Fully automated procedure, based on Gabor wavelet enhanced images, developed by Azemin et al. (2011)	Each SD decrease in baseline SFDt was associated with 40% greater risk of stroke events
McGrory et al. (2019)	**FDa, FDv (monofractal)** CRAE, CRVE BSTDa, BSTDv WGa, WGv cTORTa, cTORTv BCa, BCv LDRa, LDRv AFa, AFv	45° digital retinal camera (CRDGi; Canon USA, Lake Success, New York,USA)	0.5–2.0 DD from disc margin	VAMPIRE (School of Computing, University of Dundee, United Kingdom)	In the LBC1936 FDa accounted for 4% of the variance in WMH load In the MSS lower FDa was associated with deep WMH scores
Ong et al. (2014)	**FDa, FDv (monofractal)** CRAE, CRVE cTORTa, cTORTv BAa, BAv [Also: retinopathy signs on fundus photographs]	45° digital retinal camera (MCRS: Canon D60, Canon, Tokyo, Japan) (SEED: Canon CR-DGi with a 10D or 20D (NS) SLR backing, Canon, Tokyo, Japan)	0.5–2.0 DD from disc margin	SIVA	Decreased FDa and FDv were associated with stroke

*AFa/v, arteriolar/venular asymmetry factor; BAa/v, arteriolar/venular branching angle; BCa/v, arteriolar/venular branching coefficient; BCFDt, total box counting fractal dimension; BSTDa/v, zone B standard deviation of arteriolar/venular width; CADASIL, cerebral autosomal dominant arteriopathy with subcortical infarcts and leukoencephalopathy; CRAE, central retinal arteriolar equivalent; CRVE, central retinal venular equivalent; cTORTa/v, curvature arteriolar/venular tortuosity; DD, disc diameter; FD, fractal dimension; FDCt, total Higuchi's fractal dimension in circumferential direction; FDt/a/v, total/arteriolar/venular fractal dimension; FD0, capacity dimension; FOV, field of view; IRIS-Fractal, International Retinal Imaging Software-Fractal; LBC1936, Lotharian Birth Cohort 1936; LDRa/v, arteriolar/venular length-diameter ratio; MCRS, Multi-Centre Retinal Stroke; MRI, magnetic resonance imaging; NS, not specified; ROI, region of interest; SD, standard deviation; SEED, Singapore Epidemiology of Eye Disease; SFDt, total spectrum (or Fourier) fractal dimension; SIVA, Singapore I Vessel Assessment; VAMPIRE, Vessel Assessment and Measurement Platform for Images of the Retina; WGa/v, arteriolar/venular width gradient*.

In acute and subacute setting, studies reported decreased microvascular network complexity in lacunar stroke compared to mild cortical stroke (Doubal et al., 2010), an association between a decrease in arteriolar FD and deep white matter hyperintensity (WMH) scores in patients with a recent lacunar or cortical stroke (McGrory et al., 2019), and a general decrease in FD in ischemic stroke compared to controls without stroke (Ong et al., 2013). In contrast, Cheung et al. showed an increased FD in patients with acute lacunar stroke compared to patients with other types of acute ischemic stroke (Cheung et al., 2010).

The only case series included in this review studied retinal microvascular complexity in cerebral autosomal dominant arteriopathy with subcortical infarcts and leukoencephalopathy (CADASIL), and the authors concluded that vascular complexity was decreased but they failed to reveal a correlation with disease duration or lesion volume on magnetic resonance imaging (Cavallari et al., 2011).

## Discussion

In this review, the outcomes of fractal analysis in neurodegeneration studies show a decreased FD in various degrees and etiologies of CI, including AD and vascular dementia, as opposed to cognitively normal controls. Despite rather small sample sizes, findings are consistent with effect sizes ranging from −0.018 (Csincsik et al., 2018) to −1.33 (Cabrera DeBuc et al., 2018). The findings in studies with stroke patients are less straightforward, probably due to the heterogeneity in both study design and methodological set-up. Overall, in acute, subacute as well as in chronic settings, decreased FD seems to be associated with stroke. Differences in FD between subtypes of ischemic stroke remain unclear. The most striking inconsistency arose from two papers by Cheung et al. and Doubal et al., both focusing on FD in lacunar stroke vs. non-lacunar stroke (Cheung et al., 2010; Doubal et al., 2010). Cheung et al. reported an increased FD in lacunar stroke compared to other stroke types, whereas Doubal et al. observed a decreased FD in lacunar stroke compared to mild cortical stroke. More recent findings by McGrory et al. (2019), reporting an association between sparser arteriolar retinal network and cerebral small vessel disease, favor those by Doubal et al.

### Potential Clinical Value of Retinal Fractal Dimension in Central Neurodegeneration and Stroke

Interesting observations are being made with retinal fractal dimension. However, a number of methodological issues need to be addressed before the metric can be considered for clinical application as a reliable and non-invasive biomarker in central neurodegeneration and/or stroke. Indeed, some hurdles remain to be taken, such as uniformization of patient populations and study design. First of all, study populations were generally well characterized but heterogeneous throughout studies (e.g., ethnicity) and overall of fairly small size. Work by Cheung et al., Ong et al., McGrory et al., and Williams et al. comprised larger population samples (Cheung et al., 2013, 2014a; Ong et al., 2013; Taylor et al., 2015; Williams et al., 2015; McGrory et al., 2019). In studies focusing on cognitive dysfunction, the standardized National Institute of Neurological and Communicative Disorders and Stroke and the Alzheimer's Disease and Related Disorders Association (NINCDS-ADRDA) criteria were mostly applied for AD diagnosis, but the assessment of healthy cognitive status or CI (stage not specified) was based on a variety of tests and criteria, often complemented by brain imaging. Comparable to the importance of etiology and disease staging in CI and dementia studies, it is equally important in stroke studies to differentiate between different stroke subtypes and acute vs. chronic setting. Second, many different methodological set-ups have been implemented. Imaging devices considered mainly conventional digital retinal cameras, but the latest studies already made the first steps toward ultra-widefield SLO and OCT-A. The most often demarcated ROI, being the annulus between 0.5 and 2.0 disc diameters from the disc margin, originated from the Atherosclerosis Risk in Communities (ARIC) cohort and has been developed for use with Canon 45° retinal photographs (modified ARIC grid) (Hubbard et al., 1999). The use of a particular ROI was not well-argumented in any of the studies. Vessel segmentation was usually done semi-automatically, using custom or commercially available software packages. Agreement between different software packages appears to be poor (McGrory et al., 2018). Huang et al. showed an important effect of vessel annotations from human observers, automatic segmentation methods, various regions of interest, accuracy of vessel segmentation methods, and different imaging modalities on FD measurement, suggesting FD must be determined under very strict conditions to deliver a stable parameter (Huang et al., 2016). After image processing, automated FD calculation was performed. The FD can be determined in a monofractal or multifractal approach. The monofractal FD is a constant for all scales, whereas the multifractal FDq describes the multifractal behavior of a structure in different scales. Monofractal FD can be calculated using Higuchi's method, using a spectrum or Fourier method, or using a box counting method. Multifractal FD comprises FD_0_, FD_1_, and FD_2_: capacity dimension, entropy dimension and correlation dimension, respectively. Because of its degree of spatial complexity, the retinal arteriolar tree may represent a composite of many monofractal dimensions, making a multifractal technique better suited to characterize such arrangement (Stosić and Stosić, 2006). The monofractal dimension was usually calculated using the box counting method. The only paper calculating monofractal dimension using all three techniques, however, identified Higuchi's FD as a better predictor of stroke (Aliahmad et al., 2014). The three papers using a multifractal approach applied the generalized sandbox method (Doubal et al., 2010; Taylor et al., 2015; Cabrera DeBuc et al., 2018). According to Doubal et al., FD_0_ would be the most appropriate measure for the complexity of the retinal microvasculature, because it appeared most sensitive to small vascular changes (Doubal et al., 2010). Besides the use of different software algorithms and calculation techniques, a distinction between arteriolar, venular, and total FD should be made. All these variables in every stage toward data collection, analysis, and interpretation make it very hard to compare findings, which argues for the development of a study protocol based on comparative research, which is currently missing. Such standardization is indispensable to determine the position of FD analysis among other potential biomarkers. Additionally, many papers included in this review did not solely focus on FD, but explored a wide range of retinal vascular parameters (Tables 2, 3) without a detailed hypothesis. The comparison of many different parameters creates the problem of multiple comparisons and thus increases the likelihood of incorrectly rejecting a null hypothesis. However, only three out of 21 papers reported the use of correction (e.g., Bonferroni correction) to control for this type I error (Taylor et al., 2015; Jung et al., 2019; McGrory et al., 2019).

### Future of Retinal Fractal Dimension: Limitations and Suggestions for Further Studies

There is a need for new, simple, non-invasive, cost-effective and reliable biomarkers for risk stratification, screening purposes, early diagnosis and follow-up in neurodegenerative disorders and stroke. Prevalence of neurodegenerative disease and stroke is increasing and causes an enormous socio-economic burden worldwide (Prince et al., 2015; Feigin et al., 2017). Diagnosis is often based on clinical symptoms complemented with technical investigations such as neuroimaging. However, diagnosis is often made at a point were irreversible damage has already occurred. On top of this, many diagnostic tests have the disadvantage of being costly, invasive and imperfect. State-of-the-art technologies for ocular imaging, such as spectral-domain optical coherence tomography (SD-OCT), OCT-A and SLO, allow to visualize retinal changes at a resolution of at least an order of a magnitude higher than conventional brain imaging techniques, without the need for invasive, costly procedures or tracers, and in a well-reproducible and quantifiable manner. These techniques are increasingly being implemented as standard equipment in ophthalmological and neurological practices. The assessment of the retinal FD, and possibly additional retinal vessel metrics, could find a way into clinical practice through these imaging techniques.

Besides standardization in the assessment of retinal FD in central neurodegeneration and stroke, in-depth knowledge about FD in health and disease is required to determine and consolidate the exact position of this parameter, e.g., in screening, risk stratification, early diagnosis, follow-up. Information on changes in FD in ocular and systemic conditions affecting the retinal vasculature needs to be supplemented to identify potential confounders. In diabetes mellitus, FD has been investigated extensively. Findings remain inconclusive, with a number of studies reporting an increased FD in patients with diabetes mellitus on one hand (Cheung et al., 2009; Lim et al., 2017; Orlando et al., 2017), and quite some studies describing a decreased FD in diabetes patients on the other hand (Avakian et al., 2002; Chen et al., 2018; Popovic et al., 2018). Inconsistencies can at least partly be explained by the wide variety in study methodology. In arterial hypertension, FD has been investigated less extensively, but studies tend to point toward a decreased FD in arterial hypertension (Liew et al., 2008; Cheung et al., 2011). A valuable population is that of “healthy” elderly, representing normal aging, including prevalent cardiovascular risk factors. Very little is known about the reversibility of FD changes, leaving the question whether lifestyle changes can “restore” FD unanswered. Another question that still remains is the exact temporal relationship between retinal and cerebral vascular changes. Prospective cohort studies with a standardized imaging and FD analysis protocol and long follow-up period can offer the answers to these questions and contribute to the understanding of the pathophysiology of neurodegenerative diseases and stroke.

## Conclusion

This review provides a summary of the scientific literature regarding the association between retinal FD and neurodegenerative disease and stroke. Central nervous system disease is associated with a decreased FD, as a measure of microvascular network complexity. As retinal FD reflects the global integrity of the cerebral microvasculature (Cheung et al., 2015), it is an attractive parameter to explore. Most studies showed an association between retinal FD and neurodegeneration or stroke, but a predictive value has not been confirmed, partly due to its low specificity. The research community should strive for uniformization and standardization in retinal vessel analysis. Future research should also delineate the normal evolution of FD with age and cardiovascular health status to take the effect of confounders into account. This is required before the development of clinical applications for retinal FD can be established.

## Data Availability Statement

All datasets generated for this study are included in the article/Supplementary Material.

## Author Contributions

SL: conception and design of study. SL, AD, and JB: acquisition, analysis of data, and drafting the manuscript. SL, KV, PD, and IS: revising the manuscript critically for important intellectual content. SL, AD, JB, KV, PD, and IS: approval of the version of the manuscript to be published.

### Conflict of Interest

The authors declare that the research was conducted in the absence of any commercial or financial relationships that could be construed as a potential conflict of interest.

## Abbreviations

Ab+: beta-amyloid positive
Ab–: beta-amyloid negative
AD: Alzheimer's disease
ADCI: Alzheimer's disease cognitive impairment
AFa/v: arteriolar/venular asymmetry factor
AIBL: Australian Imaging, Biomarkers and Lifestyle
aMCI: amnestic mild cognitive impairment
AMT: Abbreviated Mental Test
AVR: artery-vein ratio
BAa/v: arteriolar/venular branching angle
BCa/v: arteriolar/venular branching coefficient
BCFDt: total box counting fractal dimension
BMES: Blue Mountains Eye Study
BSTDa/v: zone B standard deviation of arteriolar/venular width
CA: case
CADASIL: cerebral autosomal dominant arteriopathy with subcortical infarcts and leukoencephalopathy
CI: cognitive impairment
CIND: cognitive impairment no dementia
CMB: cerebral microbleeds
CO: control
CRAE: central retinal arteriolar equivalent
CRVE: central retinal venular equivalent
CSVD: cerebral small vessel disease
CT: computed tomography
cTORTa/v: curvature arteriolar/venular tortuosity
DD: disc diameter
DSM-IV: Diagnostic and Statistical Manual of Mental Disorders IV
DVP: deep vascular plexus
EDIS: Epidemiology of Dementia in Singapore
FD: fractal dimension
FDCt: total Higuchi's fractal dimension in circumferential direction
FDt/a/v: total/arteriolar/venular fractal dimension
FD0: capacity dimension (multifractal)
FD1: information dimension (multifractal)
FD2: correlation dimension (multifractal)
FOV: field of view
IQ: intelligence quotient
IRIS-Fractal: International Retinal Imaging Software-Fractal
JEa/v: junctional exponent deviation for arterioles/venules
LBC1936: Lotharian Birth Cohort 1936
LDRa/v: arteriolar/venular length-diameter ratio
LI: lacunar infarction
LOE: level of evidence
M/F: male-female ratio
MCI: mild cognitive impairment
MCRS: Multi-Centre Retinal Stroke
MMSE: Mini-Mental State Examination
MoCA: Montreal Cognitive Assessment
MRI: magnetic resonance imaging
MSS: Mild Stroke Study
NCI: noncognitive impairment
NIA-AA: National Institute on Aging–Alzheimer's Association
NINCDS-ARDRA: National Institute of Neurological and Communicative Disorders and Stroke and the Alzheimer's Disease and Related Disorders Association
NS: not specified
Num1stBa/v: number of first branching arterioles/venules
OCT-A: optical coherence tomography angiography
OD: optic disc
PD: Parkinson's disease
RVN: retinal vascular network
RVP: retinal vascular parameters
SCES: Singapore Chinese Eye Study
SD: standard deviation
SD-OCT: spectral domain optical coherence tomography
SEED: Singapore Epidemiology of Eye Disease
SFDt: total spectrum (or Fourier) fractal dimension
SiMES: Singapore Malay Eye Study
SINDI: Singapore Indian Eye Study
SIVA: Singapore I Vessel Assessment
SLO: scanning laser ophthalmoscopy
sTORTa/v: simple arteriolar/venular tortuosity
SVaD: subcortical vascular dementia
SVCI: subcortical vascular cognitive impairment
svMCI: subcortical vascular mild cognitive impairment
SVP: superficial vascular plexus
T2DM: type 2 diabetes mellitus
TICSM: Telephone Interview for Cognitive Status
TOAST: Trial of Org 10172 in Acute Stroke Treatment
UWF: ultra-widefield
VAMPIRE: Vessel Assessment and Measurement Platform for Images of the Retina
WGa/v: arteriolar/venular width gradient
WHO-MONICA: World Health Organization Monitoring Trends and Determinants in Cardiovascular Disease
WLCDTRU: West London Cognitive Disorders Treatment and Research Unit
WMH: white matter hyperintensity
WML: white matter lesions.
