# Supplementary material for: Systematic Review on Fractal Dimension of the Retinal Vasculature in Neurodegeneration and Stroke: Assessment of a Potential Biomarker
Source: Front Neurosci. 2020 Jan 28;14:16. doi: 10.3389/fnins.2020.00016 (PMC7025576; doi:10.3389/fnins.2020.00016)
Supplement: Supplementary file 1 [file Table_1.DOCX]

Supplementary Material

# Supplementary data 1: search strategy

EMBASE

((('retina'/exp OR ‘retina*’:ti,ab OR (('retina'/exp OR ‘retina*’:ti,ab ) AND (‘vascularization’/exp OR ‘vascularization*’:ti,ab OR ‘blood supply’:ti,ab)) OR ‘retina blood vessel*’:ti,ab OR ‘retinal vasculature*’:ti,ab)) AND ('fractal analysis'/exp OR ‘fractal*’:ti,ab)) AND (‘degenerative disease’/exp OR ‘degenerative disease’:ti,ab OR ‘neurodegenerative disease*’:ti,ab OR ‘dementia’/exp OR ‘dementia’:ti,ab OR ‘amentia’:ti,ab OR ‘demention’:ti,ab OR ‘cognitive defect’/exp OR ‘cognitive defect’:ti,ab OR ‘cognition disorder’:ti,ab OR ‘cognition disorders’:ti,ab OR ‘cognitive defects’:ti,ab OR ‘cognitive deficit’:ti,ab OR ‘cognitive disability’:ti,ab OR ‘cognitive disorder’:ti,ab OR ‘cognitive disorders’:ti,ab OR ‘cognitive dysfunction’:ti,ab OR ‘cognitive impairment’:ti,ab OR ‘amnestic’:ti,ab OR ‘cognitive disorders’:ti,ab OR ‘overinclusion’:ti,ab OR ‘response interference’:ti,ab OR ‘Alzheimer disease’:ti,ab OR ‘Alzheimer* disease’:ti,ab OR ‘Alzheimers disease’:ti,ab OR ‘Alzheimer dementia’:ti,ab OR ‘alzheimer fibrillary change’:ti,ab OR ‘alzheimer fibrillary lesion’:ti,ab OR ‘alzheimer neurofibrillary change’:ti,ab OR ‘alzheimer neurofibrillary degeneration’:ti,ab OR ‘alzheimer neuron degeneration’:ti,ab OR ‘alzheimer perusini disease’:ti,ab OR ‘alzheimer sclerosis’:ti,ab OR ‘alzheimer syndrome’:ti,ab OR ‘alzheimer* disease’:ti,ab OR ‘Alzheimers disease’:ti,ab OR ‘alzheimer dementia’:ti,ab OR ‘diffuse cortical sclerosis’:ti,ab OR ‘late onset alzheimer disease’:ti,ab OR ‘diffuse Lewy body disease’/exp OR ‘diffuse Lewy body disease’:ti,ab OR ‘dementia with lewy bodies’:ti,ab OR ‘dementia with Lewy body’:ti,ab OR ‘DLB’:ti,ab OR ‘LBD’:ti,ab OR ‘Lewy body dementia’:ti,ab OR ‘Lewy body dementias’:ti,ab OR ‘Lewy body disease’:ti,ab OR ‘lewy body diseases’:ti,ab OR ‘Parkinson disease’:ti,ab OR ‘paralysis agitans’:ti,ab OR ‘parkinson dementia complex’:ti,ab OR ‘postencephalitic parkinson disease’:ti,ab OR ‘secondary parkinson disease’:ti,ab OR ‘symptomatic parkinson disease’:ti,ab OR ‘Parkinson* disease’:ti,ab OR ‘Parkinsons disease’:ti,ab OR ‘frontotemporal*’:ti,ab OR 'cerebrovascular accident'/exp OR ‘cerebrovascular accident*’:ti,ab OR ‘accident,cerebrovascular’:ti,ab OR ‘acute cerebrovascular lesion’:ti,ab OR ‘acute focal cerebral vasculopathy’:ti,ab OR ‘acute stroke’:ti,ab OR ‘apoplectic stroke’:ti,ab OR ‘apoplexia’:ti,ab OR ‘blood flow disturbance, brain’:ti,ab OR ‘brain accident’:ti,ab OR ‘brain attack’:ti,ab OR ‘brain blood flow disturbance’:ti,ab OR ‘brain insult’:ti,ab OR ‘brain insultus’:ti,ab OR ‘brain ischaemic attack’:ti,ab OR ‘brain ischemic attack’:ti,ab OR ‘brain vascular accident’:ti,ab OR ‘cerebral apoplexia’:ti,ab OR ‘cerebral insult’:ti,ab OR ‘cerebral stroke’:ti,ab OR ‘cerebral vascular accident’:ti,ab OR ‘cerebral vascular insufficiency’:ti,ab OR ‘cerebro vascular accident’:ti,ab OR ‘cerebrovascular arrest’:ti,ab OR ‘cerebrovascular failure’:ti,ab OR ‘cerebrovascular injury’:ti,ab OR ‘cerebrovascular insufficiency’:ti,ab OR ‘cerebrovascular insult’:ti,ab OR ‘cerebrum vascular accident’:ti,ab OR ‘cryptogenic stroke’:ti,ab OR ‘CVA’:ti,ab OR ‘ischaemic cerebral attack’:ti,ab OR ‘ischaemic seizure’:ti,ab OR ‘ischemic cerebral attack’:ti,ab OR ‘ischemic seizure’:ti,ab OR ‘stroke’:ti,ab)

MEDLINE

((("retina"[Mesh] OR retina*[tiab] OR "retinal vessels"[Mesh] OR retinal vessel*[tiab] OR "retinal vasculature"[tiab] OR ((“retina”[mesh] OR retina*[tiab]) AND (“blood supply”[mesh] OR “blood supply”[tiab] OR “vascularization”[tiab])))) AND (“Fractals”[Mesh] OR fractal*[tiab])) AND (((“Neurodegenerative Diseases”[Mesh] OR Neurodegenerative disease*[tiab] OR Parkinson Disease*[tiab] OR "Dementia"[Mesh] OR Dementia*[tiab] OR Alzheimer disease*[tiab] OR Lewy Body Disease*[tiab] OR “Cognitive dysfunction”[Mesh] OR Cognitive dysfunction*[tiab] OR Cognitive impairment*[tiab] OR Frontotemporal*[tiab] OR “degenerative disease”[tiab] OR “cognitive disorder”[tiab] OR “cognitive disorders”[tiab] OR “cognition disorders”[tiab] OR “cognitive defects”[tiab] OR “cognitive deficit”[tiab] OR “cognitive disability”[tiab] OR “amnestic”[tiab] OR “overinclusion”[tiab] OR “response interference”[tiab] OR “alzheimer dementia”[tiab] OR “alzheimer fibrillary change”[tiab] OR “alzheimer fibrillary lesion”[tiab] OR “alzheimer neurofibrillary change”[tiab] OR “alzheimer neurofibrillary degeneration”[tiab] OR “alzheimer neuron degeneration”[tiab] OR “alzheimer perusini disease”[tiab] OR “alzheimer sclerosis”[tiab] OR “alzheimer syndrome”[tiab] OR “diffuse cortical sclerosis”[tiab] OR “late onset alzheimer disease”[tiab] OR “diffuse lewy body disease”[tiab] OR “dementia with lewy body”[tiab] OR “dementia with lewy bodies”[tiab] OR “DLB”[tiab] OR “LBD”[tiab] OR “lewy body dementia”[tiab] OR “lewy body dementias”[tiab] OR “paralysis agitans”[tiab] OR “parkinson dementia complex”[tiab] OR “postencephalitic parkinson disease”[tiab] OR “secondary parkinson disease”[tiab] OR “symptomatic parkinson disease”[tiab])) OR ("Stroke"[Mesh] OR Stroke*[tiab] OR “Brain Infarction”[tiab] OR “lacunar infarct”[tiab] OR “cerebrovascular accident”[tiab] OR “acute cerebrovascular lesion”[tiab] OR “acute focal cerebral vasculopathy”[tiab] OR “apoplexia”[tiab] OR “brain accident”[tiab] OR “brain attack”[tiab] OR “brain blood flow disturbance”[tiab] OR “brain insult”[tiab] OR “brain insultus”[tiab] OR “brain ischaemic attack”[tiab] OR “brain ischemic attack”[tiab] OR “brain vascular accident”[tiab] OR “cerebral apoplexia”[tiab] OR “cerebral insult”[tiab] OR “cerebral vascular accident” [tiab] OR “cerebral vascular insufficiency”[tiab] OR “cerebro vascular accident”[tiab] OR “cerebrovascular arrest”[tiab] OR “cerebrovascular failure”[tiab] OR “cerebrovascular injury”[tiab] OR “cerebrovascular insufficiency”[tiab] OR “cerebrovascular insult” [tiab] OR “cerebrum vascular accident”[tiab] OR “CVA”[tiab] OR “ischaemic cerebral attack”[tiab] OR “ischaemic seizure”[tiab] OR “ischemic cerebral attack”[tiab] OR “ischemic seizure”[tiab]))

Web Of Science

“retina*” OR “retinal vessel*” OR "blood supply" OR "retinal vasculature" OR ((“retina*”) AND (“blood supply” OR “vascularization”)) AND “fractal*” AND Neurodegenerative disease* OR Parkinson* OR Dementia* OR Alzheimer* OR Lewy Body* OR Cognitive dysfunction* OR Cognitive impairment* OR Frontotemporal* OR “degenerative disease” OR “cognitive disorder” OR “cognitive disorders” OR “cognition disorders” OR “cognitive defects” OR “cognitive deficit” OR “cognitive disability” OR “amnestic” OR “overinclusion” OR “response interference” OR “alzheimer dementia” OR “alzheimer fibrillary change” OR “alzheimer fibrillary lesion” OR “alzheimer neurofibrillary change” OR “alzheimer neurofibrillary degeneration” OR “alzheimer neuron degeneration” OR “alzheimer perusini disease” OR “alzheimer sclerosis” OR “alzheimer syndrome” OR “diffuse cortical sclerosis” OR “late onset alzheimer disease” OR “diffuse lewy body disease” OR “dementia with lewy body” OR “dementia with lewy bodies” OR “DLB” OR “LBD” OR “lewy body dementia” OR “lewy body dementias” OR “paralysis agitans” OR “parkinson dementia complex” OR “postencephalitic parkinson disease” OR “secondary parkinson disease” OR “symptomatic parkinson disease” OR "Stroke" OR “Brain Infarction” OR “lacunar infarct” OR “cerebrovascular accident” OR “acute cerebrovascular lesion” OR “acute focal cerebral vasculopathy” OR “apoplexia” OR “brain blood flow disturbance” OR “brain accident” OR “brain attack” OR “brain blood flow disturbance” OR “brain insult” OR “brain insultus” OR “brain ischaemic attack” OR “brain ischemic attack” OR “brain vascular accident” OR “cerebral apoplexia” OR “cerebral insult” OR “cerebral stroke” OR “cerebral vascular accident” OR “cerebral vascular insufficiency” OR “cerebro vascular accident” OR “cerebrovascular arrest” OR “cerebrovascular failure” OR “cerebrovascular injury” OR “cerebrovascular insufficiency” OR “cerebrovascular insult” OR “cerebrum vascular accident” OR “cryptogenic stroke” OR “CVA” OR “ischaemic cerebral attack” OR “ischaemic seizure” OR “ischemic cerebral attack” OR “ischemic seizure”
